# Genome-wide analysis of H3.3 dissociation reveals high nucleosome turnover at distal regulatory regions of embryonic stem cells

**DOI:** 10.1186/1756-8935-7-38

**Published:** 2014-12-20

**Authors:** Misook Ha, Daniel C Kraushaar, Keji Zhao

**Affiliations:** Samsung Advanced Institute of Technology, Samsung Electronics Corporation, Yongin-Si, 446-712 Gyeonggi-Do South Korea; Systems Biology Center, National Heart, Lung, and Blood Institute, NIH, Bethesda, MD 20892 USA

**Keywords:** Histone variant, H3.3 dissociation, Nucleosome stability, Genome-wide chromatin dynamics

## Abstract

**Background:**

The histone variant H3.3 plays a critical role in maintaining the pluripotency of embryonic stem cells (ESCs) by regulating gene expression programs important for lineage specification. H3.3 is deposited by various chaperones at regulatory sites, gene bodies, and certain heterochromatic sites such as telomeres and centromeres. Using Tet-inhibited expression of epitope-tagged H3.3 combined with ChIP-Seq we undertook genome-wide measurements of H3.3 dissociation rates across the ESC genome and examined the relationship between H3.3-nucleosome turnover and ESC-specific transcription factors, chromatin modifiers, and epigenetic marks.

**Results:**

Our comprehensive analysis of H3.3 dissociation rates revealed distinct H3.3 dissociation dynamics at various functional chromatin domains. At transcription start sites, H3.3 dissociates rapidly with the highest rate at nucleosome-depleted regions (NDRs) just upstream of Pol II binding, followed by low H3.3 dissociation rates across gene bodies. H3.3 turnover at transcription start sites, gene bodies, and transcription end sites was positively correlated with transcriptional activity.

H3.3 is found decorated with various histone modifications that regulate transcription and maintain chromatin integrity. We find greatly varying H3.3 dissociation rates across various histone modification domains: high dissociation rates at active histone marks and low dissociation rates at heterochromatic marks. Well- defined zones of high H3.3-nucleosome turnover were detected at binding sites of ESC-specific pluripotency factors and chromatin remodelers, suggesting an important role for H3.3 in facilitating protein binding. Among transcription factor binding sites we detected higher H3.3 turnover at distal cis-acting sites compared to proximal genic transcription factor binding sites.

Our results imply that fast H3.3 dissociation is a hallmark of interactions between DNA and transcriptional regulators.

**Conclusion:**

Our study demonstrates that H3.3 turnover and nucleosome stability vary greatly across the chromatin landscape of embryonic stem cells. The presence of high H3.3 turnover at RNA Pol II binding sites at extragenic regions as well as at transcription start and end sites of genes, suggests a specific role for H3.3 in transcriptional initiation and termination. On the other hand, the presence of well-defined zones of high H3.3 dissociation at transcription factor and chromatin remodeler binding sites point to a broader role in facilitating accessibility.

**Electronic supplementary material:**

The online version of this article (doi:10.1186/1756-8935-7-38) contains supplementary material, which is available to authorized users.

## Background

In eukaryotes, genomic DNA is packaged as chromatin, a complex of DNA, proteins, and RNAs. The basic unit of chromatin is the nucleosome, which consists of 147 bp DNA wrapped around histone proteins. Incorporation of histone variants into chromatin critically influences the properties of nucleosomes that play important roles in regulating transcription and epigenetic memory. Among three major histone H3 variants, H3.1, H3.2, and H3.3, H3.3 marks important DNase I hypersensitive *cis*-regulatory elements including promoters, enhancers, and insulators [1–4]. Different from canonical histone H3, which is expressed in S-phase and is incorporated into chromatin during DNA replication, H3.3 can be incorporated into chromatin independent of DNA replication [3, 5]. The incorporation of histone variants is tightly regulated by histone chaperones. The H3.3-specific chaperones Atrx-Daxx and HIRA deposit H3.3 primarily at telomeres and non-heterochromatic regions, respectively [1, 6, 7].

H3.3 plays important regulatory roles in both transcriptional activation and repression. Upon gene activation, H3.3 is incorporated into transcription start sites (TSSs) and coding regions of genes, where it continues to be deposited even when transcription ceases [8, 9].

Histone marks that are associated with gene activation, such as acetylation marks and H3K4me3, are typically found on H3.3 whereas marks associated with gene silencing such as H3K27me3 and H3K9me3 are predominantly found on H3.1 and H3.2 [10]. The precise mechanism by which H3.3 promotes gene activation is not known but may involve the destabilization of the nucleosome particle upon incorporation [2, 11]. On the other hand, H3.3 facilitates the recruitment of Polycomb proteins in ESCs and establishment of H3K27me3 domains that are associated with gene silencing [11]. Moreover, H3.3 is required for heterochromatin formation at pericentromeric regions and telomeres in mouse embryonic stem cells [7, 12].

Histone and nucleosome turnover have been studied extensively in yeast, where a single histone variant H3.3 is expressed. More recently, we reported genome-wide histone H3.3 turnover in mouse embryonic fibroblasts and showed that the dynamics of histone turnover appear to be largely conserved among eukaryotes [13]. Our results revealed three major categories of H3.3 nucleosome turnover: rapid turnover at enhancers and promoters, intermediate turnover at gene bodies, and slow turnover at heterochromatic regions. However, the H3.3 turnover was inferred from the incorporation rate of newly synthesized H3.3 and the dissociation of H3.3 from chromatin has not been directly observed.

Embryonic stem cells (ESCs) are characterized by their pluripotent differentiation capacity that is manifested by a unique chromatin structure. ESCs carry an open chromatin configuration with a large number of bivalent genes that are marked by both active and repressive marks, which resolve upon lineage commitment [14–16]. Unlike in differentiated mammalian cells, where the pattern of H3.3 enrichment is associated primarily with gene activity, H3.3 is detected at numerous promoters of inactive genes in ESCs underlining once more the unique chromatin state of ESCs [1]. Furthermore the ESC chromatin displays a hyper-dynamic state in which chromatin components including histones and even heterochromatic proteins exhibit rapid exchange compared to differentiated somatic cells [17]. Hence understanding the histone dynamics of ESCs not only on a global but also a genome-wide scale is of utmost interest.

Genome-wide histone turnover has traditionally been studied using inducible expression systems that include epitope-tagged histones or, alternatively, metabolically labeled histones that are immuno-precipitated by ChIP and subsequently subjected to deep sequencing techniques. TET-inducible histone expression combined with ChIP-Seq typically result in initial increases, followed by decreases in read density over many genomic regions due to read coverage limitations [13, 18, 19]. Therefore, calculations of turnover with linear regression models are inevitably difficult to perform when induction systems are utilized.

In this study, we set out to examine histone dynamics by measuring the dissociation rates of previously incorporated histones. To this end, we generated a Tet-OFF ESC line that expresses HA/FLAG-tagged H3.3 and allowed us to track the dissociation of H3.3 upon TET-induced inhibition of H3.3 expression. Using an analytical approach that quantifies the dissociation rates of H3.3 in high resolution, we find that the H3.3 dissociation rate from chromatin varies distinctly across the ESC genome.

Our analyses identified distinct dynamics of H3.3 as they relate to transcriptional regulation and heterochromatin integrity. Transcription initiation and end sites are marked by very high dissociation rates of H3.3, that correlate well with gene activity. In contrast, inside coding regions H3.3 equilibrium levels are low and display low turnover. H3.3 dissociation rates were found to differ greatly depending on post-translational modifications with higher dissociation rates associated with active marks and lower rates at heterochromatic marks. On the other hand, binding sites of pluripotency factors and chromatin remodelers were marked by invariably high turnover rates. Surprisingly, we found that distant-acting regulatory sites are dynamically regulated by higher rates of H3.3 dissociation than we found at proximal regulatory sites. Therefore we conclude that distinct H3.3 turnover rates provide a molecular mechanism to accommodate chromatin architecture, transcription as well as accessibility of ESC specific transcription factors and chromatin remodelers.

## Results

### Genome-wide measurement of H3.3 dissociation rates with a ‘TET-OFF’ ESC line

**Figure 1:**
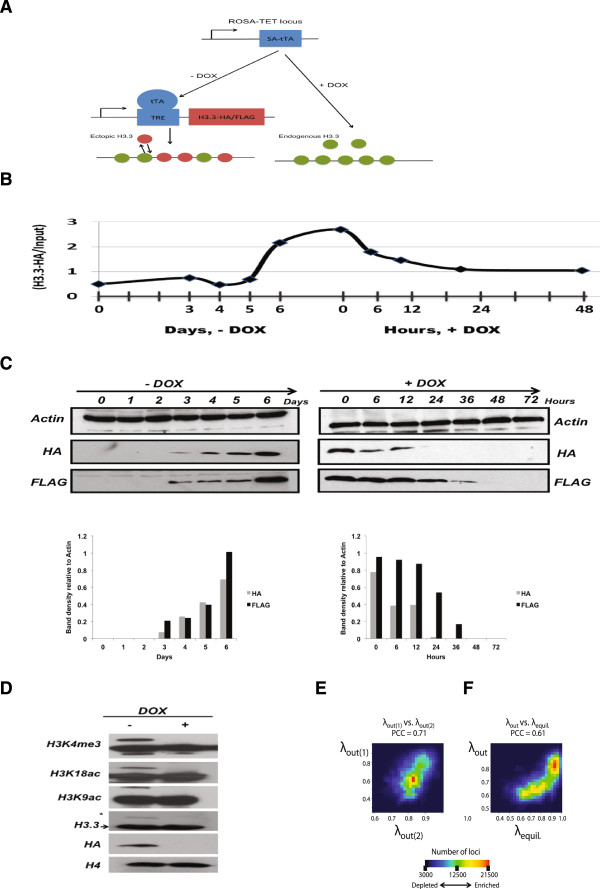
**Experimental design to measure genome-wide dissociation rates of newly synthesized H3.3-HA in mouse embryonic stem cells. (A)** Schematic of TET-repressible HA-H3.3 expression system. Green and red circles represent endogenous and newly synthesized H3.3, respectively. Ectopically expressed HA-H3.3 incorporates into chromatin over time and reaches an equilibrium level before addition of doxycycline inhibits HA-H3.3 expression. Upon inhibition of its expression, HA-H3.3 dissociates from chromatin over time. **(B)** Average enrichment of HA-H3.3 ChIP-Seq reads over time. ESCs were cultured for multiple passages with 2 ug/mL DOX before DOX was removed and HA-H3.3 expression induced over a time course of 6 days. ESCs routinely cultured without DOX were exposed to 2 μg/mL DOX to inhibit HA-H3.3 expression. Steady-state levels of HA-H3.3 enrichment (tpnt. 0 h) decline over a time course of 48 h. Horizontal axis depicts time in days during induction of HA-H3.3 expression (- DOX condition) and time in hours during repression (+ DOX condition) of HA-H3.3 expression. **(C)** Time course western blots showing expression of HA-H3.3 during induction and inhibition of HA-H3.3 expression. ESCs were treated with or without 2 μg/mL DOX. Densitometry was calculated using the Image J software package. **(D)** Western blot showing comparison between endogenous H3.3 and ectopic HA-H3.3 expression. The asterisk marks transgenic HA-H3.3; the arrow marks endogenous H3.3. **(E)** Density blot showing the correlation of HA-H3.3 dissociation rates measured in 10 bp windows between two biological replicate experiments. The color represents number of 10 bp windows with highest points (red) and lowest points (dark blue). Pearson’s correlation coefficient (PCC) between two H3.3 dissociation rates is 0.71, *P* value = 0, df = 6836487. **(F)** H3.3 dissociation rates are highly correlated with its equilibrium levels. Density blot showing correlation between H3.3 dissociation rates and H3.3 equilibrium levels. PCC = 0.61, df = 6836487, *P* value = 0.

To measure dissociation rates of H3.3, we utilized a TET-repressible ESC line, ES(MC1R(20)), with the expression cassette integrated at the ROSA26 locus [20]. We transfected MC1R ESCs with HA/FLAG-tagged H3.3 controlled by tetracycline response elements. ESCs that were routinely cultured in the absence of DOX were exposed to DOX-containing medium in order to inhibit HA-H3.3 expression and to measure H3.3 dissociation rates (λ_out_) (Figure 1A). This ectopic expression system was combined with ChIP-Seq to measure HA-H3.3 dissociation from chromatin over time. The HA-H3.3 ESC line maintained high alkaline phosphatase activity and a dome-shaped colony morphology on both gelatin-coated dishes and feeder cells, which was not affected by tagged H3.3 expression in the absence of DOX. In addition, ESC clones formed alkaline phosphatase positive colonies from single cells and were similar in size between cells that expressed HA-H3.3 and those that did not, suggesting that transgene expression did not interfere with ESC self-renewal and proliferation (Additional file 1: Figure S1 A). Furthermore, HA-H3.3 ESC maintained their pluripotent state as demonstrated by the high expression of the pluripotency genes Oct4, Nanog and Sox2, which became downregulated upon embryoid body (EB) differentiation (Additional file 1: Figures S1 B and C). At the same time, markers of the endoderm and mesoderm lineages, Sox17 and Kdr, respectively, were robustly upregulated during EB formation in the presence and absence of DOX (Additional file 1: Figure S1 D).

ESCs that had been routinely cultured in the presence of DOX displayed upregulation of HA-H3.3 over several days following removal of DOX. ESC cultures that had not been previously exposed to DOX, expressed steady-state levels of HA-H3.3 (time point 0 h, λ_equ_). Addition of DOX resulted in the rapid downregulation of HA-H3.3 after 6 h and HA-H3.3 levels continued to decline over a time course of 48 h (Figures 1B and C). Immunoblotting against H3.3 revealed that transgenic H3.3 was expressed at low levels compared to endogenous H3.3 and that C-terminus HA and FLAG tags did not interfere with posttranslational modification of H3.3 (Figure 1D).

To analyze H3.3-nucleosome dynamics at any given locus we measured dissociation rates (λ_out_) as the change of reads from time point 0 h to time point 6 h. We validated the ChIP-Seq read enrichment and decline of HA-H3.3 at two control regions by ChIP-PCR. ChIP-Seq profiles revealed high enrichment of HA-H3.3 at the 3’UTR of Rps19 and depletion of HA-H3.3 at an intergenic region on chromosome 8. ChIP-PCR experiments confirmed enrichment at Rps19, which declined over the time course of DOX addition (Additional file 2: Figure S2).

Measurements of dissociation rates were highly reproducible between replicate experiments at high resolution (10 bp) (Pearson’s correlation coefficient (PCC) = 0.712, *P* value = 0, df = 6836487) (Figure 1E). We further examined the overall relationship between H3.3 dissociation rates and equilibrium enrichment levels. Among loci significantly enriched with HA-H3.3, H3.3 dissociation rates were significantly correlated with equilibrium levels of H3.3 (PCC, λ_*out*_ vs. λ_*equil.*_ = 0.61, *P* value = 0, df = 6836487) (Figure 1F), suggesting that H3.3 deposition and high nucleosome dissociation are intrinsically linked.

### High H3.3 dissociation rates mark RNA Pol II bound sites of transcription

**Figure 2:**
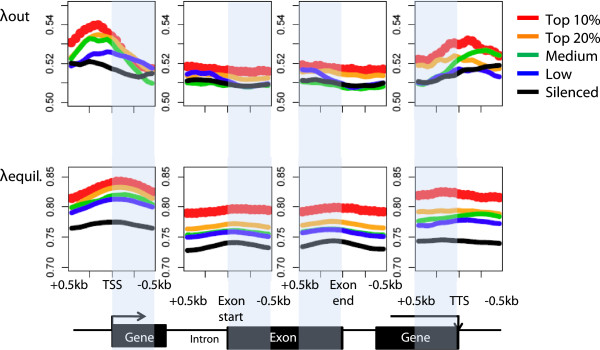
**H3.3 dissociation rates are positively correlated with gene activity.** RNA-Seq data were used to group genes according to transcription levels based on their FPKM values into Top 10% (>30 FPKM) and Top 20% (10 to 30 FPKM), medium (3 to 10 FPKM) and low (1 to 3 FPKM) and silent (<1 FPKM) genes. H3.3 dissociation rates and equilibrium levels were measured in a 1 kb window around transcription start sites, exon start sites, exon end sites and transcription termination sites. Dissociation rates were calculated based on changes in HA-H3.3 enrichment from time point 0 h to time point 6 h after DOX addition. Gray shading marks coding regions.

Next, we examined whether H3.3 dissociation rates are affected by gene transcription levels. Based on our RNA-Seq data derived from undifferentiated HA-H3.3 ESCs, we categorized genes according to their transcription levels. At transcription start and end sites, H3.3 dissociation rates and equilibrium levels were positively correlated with transcription levels (Figure 2). These findings suggest that the rapid dissociation of H3.3 is associated with efficient transcription initiation and termination. We detected significant albeit low enrichment of H3.3 at TSSs of silent genes. This suggests that once H3.3 is incorporated at silenced genes, it is stably maintained and shows low dissociation rates. At the TSS of actively transcribed genes, H3.3 deposition is associated with high dissociation rates and suggests that rapid H3.3 turnover facilitates the transcription process.

Within coding regions, both H3.3 enrichment and dissociation rates are significantly correlated with transcription levels, which further supports the idea that both H3.3 deposition and dissociation are a direct function of Pol II- dependent transcription. However, the relative rate of dissociation was substantially lower within gene bodies as compared to TSSs and transcription end sites (TESs), suggesting that distinct modes of H3.3 deposition and eviction exist at coding regions and transcription start and end sites.

H3.3 equilibrium levels and dissociation rates were equal at exon-intron junctions relative to other regions within the coding region and therefore H3.3 exchange may not be involved in splicing processes.

**Figure 3:**
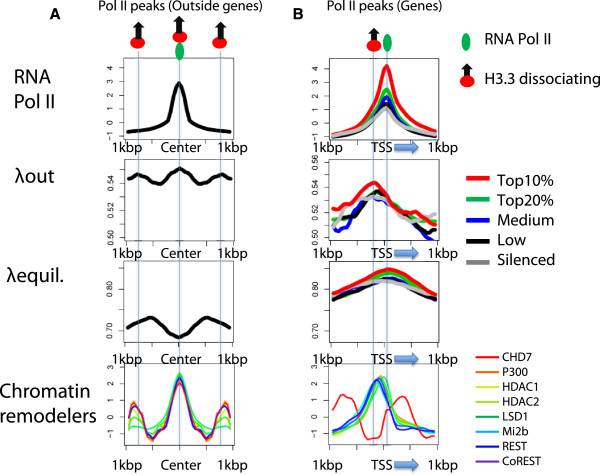
**High H3.3 dissociation rates mark RNA Pol II binding sites. (A)** H3.3 dissociation rates and equilibrium levels were measured at non-genic Pol II binding sites. Green, red, and blue colored ovals indicate Pol II binding, dissociating, and incorporating H3.3 nucleosomes at the sites. Pol II, CHD7, p300, HDAC1, HDAC2, LSD1, Mi2b, REST, and CoREST enrichment (indicated on the right of the figure) was mapped from published ChIP-Seq data (see Materials and Methods). The enrichment levels of RNA Pol II and individual chromatin remodelers were standardized to yield a distribution with mean = 0 and variance = 1 using ChIP-Seq read numbers. All plots are calculated by averaging values of the 100 bp sliding windows of the corresponding loci. **(B)** H3.3 dissociation rates and equilibrium levels at genic Pol II peaks. RNA-Seq libraries were used to group genes according to their transcription levels based on their FPKM values as indicated on the right. Blue arrows indicate the direction of transcription.

Extragenic transcription by RNA Pol II generates various types of short and long non-coding RNAs. Seventy percent of non-coding RNAs are represented by short enhancer-derived RNAs (eRNAs) that are transcribed in both directions [21, 22]. At genes, RNA Pol II generates transcripts in one direction. On transcription initiation, Pol II-associated factors create a transcription bubble and the synthesis of 25 nt RNA facilitates the transition into a stable elongation complex [23]. To investigate the relationship between RNA Pol II elongation and H3.3 turnover, we separately measured H3.3 eviction and steady-state levels of H3.3 at RNA Pol II peaks in genic regions and outside of genes. Outside of known transcribed genes, Pol II binding sites are marked by high dissociation rates and low enrichment levels of H3.3 nucleosomes. In addition, these binding sites are flanked by high peaks of H3.3 dissociation and enrichment levels (Figure 3A). The deposition of H3.3 at adjacent regions of RNA Pol II binding sites may impede Pol II or accommodate the binding of termination factors and result in short transcripts at enhancer elements and thereby ultimately define transcription end sites outside of genes.

Within a 10 kbp interval of the genic region, the highest Pol II peaks are detected within a 1 kbp region around TSSs and are positively correlated with gene expression levels. We then compared our measurements of H3.3 dissociation with RNA Pol II enrichment across the direction of transcription. The highest enrichment levels of H3.3 around Pol II binding sites are measured exactly at the binding peak of RNA Pol II. Interestingly, the peak of highest dissociation occurred around 50 to 100 bp upstream of the TSSs, which is typically nucleosome-depleted and located between the -1 and +1 nucleosome (Figure 3B).

NuRD (nucleosome remodeling and histone deacetylase) is a complex of ATP-dependent chromatin remodelers, histone methylases, demetylases, and deacetylases [24]. The NuRD subunits include CHD proteins, Mi-2b, HDAC1, HDAC2, and LSD1. Examination of NuRD subunit binding around RNA Pol II peaks show that NuRD components are highly associated with regions of high H3.3 dissociation (Figures 3A and B). Hence, NuRD nucleosome remodeling may directly destabilize H3.3 nucleosomes at transcription start sites but further investigation is needed. All of these results suggest that H3.3 deposition as well as H3.3 turnover are important spatial determinants of RNA Pol II-dependent transcription and directionality.

### H3.3 turnover associated with histone modifications and regulatory sites

#### Histone modifications

H3.3 is a carrier of various post-translational histone modifications that influence gene transcription. To test if histone modifications affect nucleosome stability, we examined H3.3 dissociation rates at sites of several histone modification domains.

**Figure 4:**
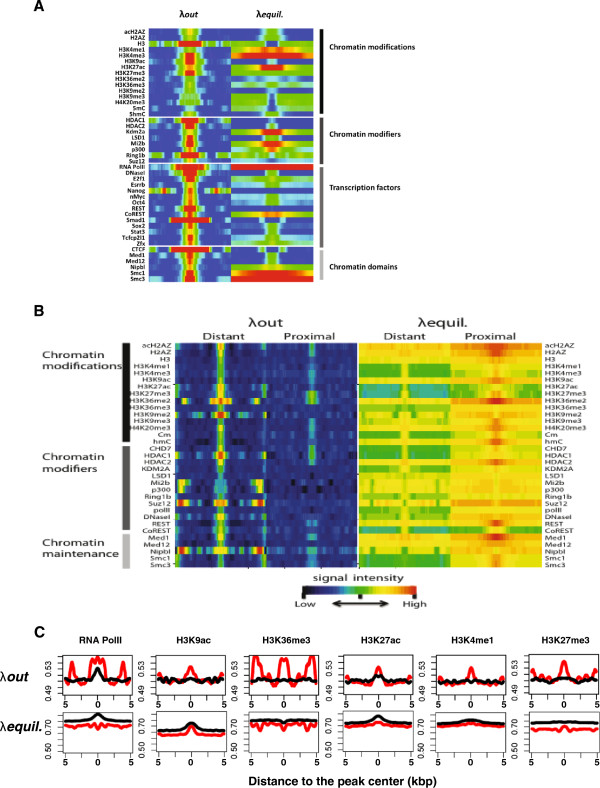
**H3.3 dissociation at regulatory elements of mouse embryonic stem cells. (A)** Heatmap illustrating the dissociation rates and equilibrium levels of H3.3 around chromatin modifications and protein binding sites. Average H3.3 dissociation rates and equilibrium levels within 10 kbp regions around sites of histone modifications and binding factors are represented with a color gradient (Red: high; Blue: low). **(B)** High H3.3 dissociation rates mark distal regulatory regions. Comparison of H3.3 dissociation rates between gene proximal and distal regulatory sites. Heatmaps showing dissociation rates and equilibrium levels of HA-H3.3 at genic and inter-genic regulatory sites. Protein binding sites located within 5 kbp of known transcripts and coding regions were considered proximal (genic) and those outside were considered distal (inter-genic). **(C)** Comparison of H3.3 dissociation rates at chromatin modification peaks between genic and inter-genic sites. Red and black lines denote inter-genic and genic values respectively

We detected the highest levels of H3.3 deposition at sites associated with active histone marks including H3K4me1, H3K4me3, H3K9ac, and H3K27ac (Figure 4A). H3.3 dissociation rates varied greatly among histone modifications: we measured the highest dissociation rates at euchromatic marks such as H3K4me3, H3K9ac, and H3K27ac. Moderate H3.3 dissociation rates were recorded at sites associated with H3K4me1 and H3K36me2/3. Only low H3.3 dissociation rates and low H3.3 equilibrium levels were measured at sites of heterochromatic histone modifications such as H3K9me2/3 and H4K20me3. Despite being associated with gene repression, we found H3K27me3 islands as well as binding sites of H3K27 methyltransferases (Ring1b, Suz12) to be sites of high H3.3 dissociation rates. Hence, although silent genes are generally found to carry low H3.3 dissociation rates, those marked by H3K27me3 exhibited exceptionally high H3.3 dissociation rates.

In gene bodies, both H3.3 enrichment and turnover are lower than at TSSs and TESs. H3K36 methylation is enriched in gene bodies and displays low dissociation rates as well as low equilibrium levels of H3.3. The result is consistent with the role of H3K36 methylation in nucleosome stabilization and inhibition of cryptic transcription [25, 26].

Heterochromatic chromatin that contains 5mC, H3K9me2/me3, H4K20me3 generally displays low H3.3 dissociation rates implying high chromatin stability (Figure 4A). Comparison of H3.3 dissociation rates at sites enriched with 5mC and 5hmC sites shows that both equilibrium and dissociation rates of H3.3 are significantly higher at 5hmC peaks (Figure 4A). 5hmC is generated by the activity of TET dioxygenases and is enriched in highly transcribed genes, Polycomb bound promoters and distal *cis*-regulatory elements [27, 28]. Our observations suggest that cytosine conversion from 5mC to 5hmC is accompanied by an increase in deposition of H3.3 that undergoes rapid nucleosome exchange. The H2A variant H2A.Z has been demonstrated to be important for ESC pluripotency and nucleosome stability [2, 29, 30]. We found strong overlap between H2A.Z and H3.3 in line with the reports that have detected both variants combined together in nucleosomes. Interestingly, we find lower stability in the acetylated form of H2A.Z compared to pan H2A.Z (Figure 4A), suggesting that the acetylated orm of H2A.Z induces lower nucleosome stability when paired with H3.3.

#### Pluripotency factors, chromatin remodelers, and chromatin organizers

ESC-specific pluripotency factor binding sites including those of Nanog, Smad1, Oct4, E2F1, Esrrb, Stat3, Tcfcp2l1, and Zfx all display high H3.3 dissociation rates with low to moderate enrichment of H3.3 in the equilibrium state (Figure 4A), suggesting that rapid H3.3-nucleosome turnover is a feature of transcription factor binding sites.

At the binding sites of chromatin remodelers and histone modifiers, such as HDAC1/2, Ezh2, LSD1, REST, CoREST, Suz12, Mi2b, and p300, various levels of H3.3 were detected with the highest deposition of H3.3 at Mi2b, Kdm2a, and p300 sites. Regardless of H3.3 equilibrium levels, H3.3 dissociation rates were found to be invariably high compared to low H3.3 turnover at regions flanking binding sites of chromatin remodelers. The acetyl transferase p300 adds H3K27ac at active enhancers. Combined with p300 we found high levels of H3.3 incorporation at chromatin regions with enhancer marks including H3K27ac and H3K4me1, demonstrating that high H3.3 dissociation is a hallmark of enhancers. Kdm2a is H3 demethylase and specifically demethylates H3K36me1 and H3K36me2 associated with centromeres where it represses transcription of small non-coding RNAs that are encoded by clusters of satellite repeats at the centromere [31]. Hence, the low H3.3 dissociation that we typically detected at heterochromatin may increase when chromatin binding needs to be accommodated. Another example of this unusually high H3.3 dissociation rate in heterochromatin is found at binding sites of the cohesin proteins Smc1/3. Smc1/3 are implicated in sister chromatin cohesion and their binding sites contained high and broad islands of H3.3 that exhibit high H3.3 turnover. In general, we find differential H3.3 dissociation rates at histone modifications and invariably high turnover at binding sites of transcription factors, chromatin remodelers, and chromatin organizers.

#### H3.3 dissociation is higher at distal regions than at proximal promoters

The histone variant H3.3 is deposited at gene promoters and also at genic and intergenic enhancers. We therefore examined whether general trends of dissociation pertain to H3.3 that is detected at genic regions compared to H3.3 that is detected at distal intergenic regions.

To this end, we separately measured H3.3 dissociation rates and equilibrium levels at chromatin modification and protein binding sites within and outside 5 kbp regions of annotated genes. Surprisingly, we find that both H3.3 dynamics and equilibrium levels are dramatically different between genic and intergenic regions. At distal regulatory regions that are marked by histone modifications and transcription factors, H3.3 steady-state levels are substantially higher in proximal regions compared to distal regions. H3.3 dissociation rates on the other hand, are significantly higher at distal binding sites (Figures 4B and C). For example, outside of coding regions, H3K36 methylation is associated with high H3.3 turnover contrary to low H3.3 dynamics inside of coding regions. The results further support that high H3.3 dynamics are associated with the regulation of interactions between proteins and DNA segments, and also suggest that promoters may maintain a slower H3.3 nucleosome exchange to accommodate the process of transcription and/or the assembly of the bulky transcription machinery may slow down the histone exchange.

### H3.3 dissociation dynamics at repeat elements

**Figure 5:**
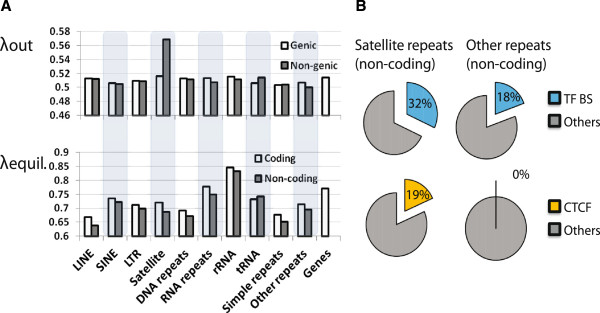
**Satellite repeats outside of genic regions exhibit high H3.3 dissociation rates and are enriched with transcription factor binding sites. (A)** H3.3 dissociation rates and equilibrium levels in various genic (white bars) and inter-genic (gray bars) repeat elements. **(B)** ChIP-Seq data (see Materials and methods) were used to map transcription factor and CTCF enrichment within various repeat elements. Pie charts depict comparisons of transcription factors and CTCF binding sites between satellite and other repeat elements located outside the genic regions.

A number of transcription factors utilize repeat elements as a major source of binding sites [32]. Therefore, we examined H3.3 dissociation rates and equilibrium levels within various repeat elements. We further tested if the H3.3 dissociation rates in repeat elements are affected by their proximity to coding regions and transcription factor binding. Repeat elements that fall into coding regions and those that do not were separately analyzed: repeat elements detected within 2 kb of a known transcript were treated as ‘genic’ repeats, others as ‘non-genic’. Satellite repeat elements located outside of genic regions show a significantly higher dissociation rate of H3.3 than other repeat elements (statistical test) (Figure 5A). We examined whether the observed differential H3.3 dissociation rates among repeat elements are affected by the binding of transcription factors. We utilized Nanog, Oct4, Klf4, E2d1, Esrrb, STAT3, Smad1, Sox2, Tcfcp2l1, and n-Myc ChIP-Seq data generated from mESCs to identify their binding sites within repeat elements. Among repeat elements, with the exception of satellite repeat elements in the non-coding region, 19% of elements are bound by at least one ESC-specific transcription factor (Nanog, Oct4, Klf4, E2d1, Esrrb, STAT3, Smad1, Sox2, Tcfcp2l1, and n-Myc). Among 2,610 satellite repeat elements located in the non-coding region, 32%, or 835, elements are significantly enriched with transcription factor binding (Chi-squared = 206,110.5, degree of freedom = 1, *P* value <10^-16^). In all cases of individual transcription factors, satellite repeats in the non-coding region showed a higher (more than two-fold) enrichment than the other repeat elements. Specifically, the number of Klf4, Esrrb, STAT3, Oct4, and Sox2 binding sites are 14.3%, 10.3%, 10.3%, 7.3%, and 7.8%, respectively, and only 2.1%, 4.8%, 4.6%, 2.8%, 3.2%, respectively, in other non-genic repeat elements (Figure 5B). The data show that higher dissociation rates of H3.3 in satellite repeat elements outside the coding region may result from or facilitate interactions between DNA and protein factors. These results further support our conclusion that high H3.3 turnover marks active interactions between DNA and protein factors. Furthermore, satellite repeat elements were shown to contain distant regulatory elements that further diversify the ESC regulatory network.

## Discussion

Histone variants have received great attention due to their important roles in transcriptional regulation. Yet, despite the availability of genome-wide maps for most histone variants, their dynamics of deposition and eviction are not fully understood. Embryonic stem cells (ESCs) are characterized by their pluripotent differentiation capacity that is manifested by a unique hyper-dynamic chromatin architecture [17]. The histone variant H3.3 is present at many regulatory sites in the ESC genome and is important for the establishment of Polycomb-mediated H3K27 methylation [11]. Here we provide not only genome-wide steady-state levels of H3.3 but also direct measurements of H3.3 dissociation rates from chromatin in mouse ESCs. Our study of H3.3 dissociation allowed us to infer important genome-wide trends of H3.3 turnover at chromatin features that are present in all mammalian cell types but also sites that are bound by ESC specific factors. We find high H3.3 dissociation rates at RNA Pol II bound genic and extragenic sites and lower dissociation rates within gene bodies. Histone H3.3 dissociation was variable among histone modification domains with high H3.3 dissociation at active histone marks and low dissociation at heterochromatic marks. Pluripotency factor and chromatin enzyme binding sites were marked by invariably high H3.3 dissociation. Interestingly, distal enhancer sites exhibited higher H3.3 turnover than gene-proximal regulatory sites. These novel results demonstrate that H3.3 nucleosome exchange is likely an important factor that functions in regulating transcriptional processes, DNA accessibility, and chromatin integrity.

### H3.3 dissociation and transcription

Active gene promoters have a highly phased structure of nucleosomes that are dynamically exchanged to allow assembly of the transcriptional machinery [33]. Nucleosomes are evicted upon transcription of chromatin and reassembled behind the progressing RNA Pol II. H3.3 deposition in gene bodies is directly coupled to RNA Pol II-mediated transcription and is executed in a gap-filling fashion [34]. When H3.3 dissociation was examined at coding regions, we found lower H3.3 dissociation within gene bodies than at promoters and 3’ ends of genes. This phenomenon has been reported from yeasts and other mammalian cells, and increased nucleosome stability within coding region has been proposed as a mechanism to prevent cryptic transcription [13, 18]. In contrast, high H3.3 dissociation at promoters and transcription end sites may facilitate transcription initiation and cessation. We found that both equilibrium levels as well as dissociation rates within TSSs, coding region, and TESs are directly correlated with gene activity. Hence, nucleosome deposition and eviction may to a great extent be a function of transcription and be dependent on binding and activity of Pol II and the transcriptional machinery. We detected highest dissociation of H3.3 nucleosomes 50 to 100 bp upstream of the RNA Pol II binding peak and peaks of dissociation overlapped tightly with binding of chromatin remodelers. Gene promoters carry a remarkably conserved and uniform nucleosome organization with well-localized -1 and +1 nucleosomes that flank so-called nucleosome-depleted regions (NDRs) just upstream of the TSSs. This pattern has been described in yeast, flies, and humans [33, 35, 36]. Hence, our mapping of H3.3 dissociation rates across promoters suggests that NDRs are in fact localized zones of extremely high H3.3 turnover. Both *cis* and *trans* factors are thought to determine nucleosome positioning and exchange [37, 38]. Chromatin remodelers have an ATPase domain that can utilize the energy of ATP to slide nucleosomes laterally or eject them entirely. Our observations that that localized regions of high H3.3 at RNA Pol II binding sites coincide with binding sites of NuRD complex components implies that this class of chromatin remodelers may directly influence the eviction of nucleosomes at TSSs.

Unexpectedly, we found that non-genic regions of highly dynamic H3.3 within 1 kbp from the nearest Pol II binding site overlap tightly with chromatin remodelers. The presence of H3.3 at these RNA Pol II flanking sites is curious and may mark TESs of non-coding RNAs.

H3.3 is a carrier of various histone modifications, but is mostly modified with active histone modifications [10]. In line with our previous study, we find high dissociation associated with active histone marks and low dissociation of H3.3 at sites marked by heterochromatic marks. The implications for the observations are two-fold. First, histone modifications themselves may determine histone stability. Histone acetylation, for instance, may through charge neutralization, disrupt DNA-histone interactions [39]. Second, heterochromatin is composed of inherently stable nucleosomes that are exchanged only at a slow rate. However, even heterochromatic sites displayed localized zones of high H3.3 dissociation when chromatin accessibility must be accommodated for instance at centromeres bound by chromatin remodelers such as Kdm2a or cohesin proteins.

Our analysis of binding sites of ESC specific factors, chromatin remodelers, and structural chromatin organizers revealed that, regardless of H3.3 equilibrium levels, H3.3 dissociation is invariably high at protein factor binding sites and drops sharply where these factors are absent. Hence, high H3.3 dissociation is a better predictor of protein factor binding than is H3.3 enrichment. At satellite repeats we detected substantially higher H3.3 dissociation than we did at other repeat elements. The elevated H3.3 dissociation was correlated with a substantially higher proportion of pluripotency factor binding within satellite repeats. Hence, the binding of ESC specific factors appears to be a strong determinant of H3.3-nucleosome turnover in ESCs. Nucleosomes are generally thought to impede transcription factor binding to their DNA motives, although some pioneer factors may bind directly to nucleosomes [40–43]. Hence, high H3.3 turnover may either be a prerequisite for transcription factor binding or be a consequence of sterical displacement by transcription factors that compete with nucleosomes for binding sites.

Interestingly, we found higher H3.3 dissociation rates at distal non-genic sites compared to proximal sites around TSSs. At the same time, equilibrium levels of H3.3 were higher at proximal H3.3 sites when compared to distal H3.3 sites.

Lower H3.3 turnover at genic regions may partially be explained by gene body H3.3, which is typically exchanged at a low rate. Yet, several marks and protein factor binding sites that are not associated with gene bodies also display higher H3.3-nucleosome turnover at distal sites compared to nucleosomes at proximal sites. Higher distal H3.3 turnover may result from H3.3-H4 tetramer splitting events, which have been reported at higher rates from enhancers compared to non-enhancers [44].

In summary, our analysis of equilibrium levels and dissociation rates in mouse ESCs revealed different modes of H3.3 deposition and turnover that pertain to their pluripotency. We detected differential trends of H3.3-nucleosome turnover as they relate to Pol II-dependent transcription, pluripotency factor binding, and proximity to genes.

## Conclusion

In this study, we utilized a TET-OFF ESC line to measure dissociation rates of H3.3 nucleosomes upon inhibition of epitope-tagged H3.3 expression. Genome-wide analyses revealed differential H3.3 turnover at functionally distinct genomic regions. High H3.3 turnover was associated with genic and extragenic sites of Pol II binding sites. At genic sites H3.3 turnover was directly correlated with gene activity suggesting that H3.3 displacement and deposition may be direct functions of transcription. Pluripotency factor binding sites as well as binding sites of chromatin enzymes displayed high H3.3 turnover even when present at heterochromatic sites, which typically feature slow H3.3 turnover, suggesting that both histone modifications and protein-DNA interactions are important determinants of nucleosome turnover. Distal regulatory sites are featured by higher turnover than gene proximal sites suggesting that the chromatin of enhancers must be kept in a highly dynamic state in order to facilitate cell type specific gene activation.

## Methods

### Generation, culture, and differentiation of HA-H3.3 ESC line

ES(MC1R(20)) cells were a kind gift from Minoru Ko. The plvx-Tight-Puro-HA/FLAG H3.3 expression construct was generated as described in [13]. Lentiviral particles were packaged in 293 T cells with the psPAX2 packaging plasmid. Subsequently, we transduced ES(MC1R(20)) cells and drug-selected with puromycin for stable integration. ESCs were cultured as in [45]. Briefly ESCs were maintained in medium consisting of Dulbecco’s modified Eagle’s medium (Hyclone) supplemented with 10% fetal bovine serum (FBS), 10% knockout serum replacement (Invitrogen), L-glutamine, 100 units/mL penicillin, 100 μg/mL streptomycin, 0.1 mM beta-mercaptoethanol at 37°C under 5% CO2, supplemented with 1,000 units/mL leukemia inhibitory factor (LIF) (ESGRO, Chemicon). For differentiation into embryoid bodies (EBs), ESCs were trypsinized and transferred to ultra-low attachment plates into 15% FBS containing medium without the addition of LIF and medium was changed every second day.

### Alkaline phosphatase assay

ESC cultures were seeded at clonal density and cultured for 5 days in ES medium (+/-2 μg/mL DOX), or directly fixed when cultured on feeder cells. Cells were washed, fixed in 4% paraformaldehyde and tested for alkaline phosphatase (AP) activity using an AP kit (Millipore).

### Quantitative PCR analysis

Total RNA was isolated using the RNeasy kit (Quiagen) and cDNA was made from 1 μg of total RNA using the SuperScript III First-Strand Synthesis System (Invitrogen). RT-PCR was performed with the following Taqman probes: Gapdh, Mm03302249; Oct4, Mm03053917; Nanog, Mm01617763; Sox2, Mm00488369; AFP, Mm00431715; Sox17, and Mm00488363.

### Western blotting

Cells were lysed with RIPA buffer, and whole-cell lysates were resolved on SDS-PAGE, transferred onto nitrocellulose membranes, and blotted with anti-HA (Roche, Basel, Switzerland), anti-FLAG (M2, Sigma, St. Louis, MO, USA) antibodies at 1:1,000. For anti-H3.3 western blotting, histones were isolated by acid extraction as described in [46] prior to immunoblotting with an anti-H3.3 antibody (Abcam, ab62642, Cambridge, UK). The anti-H3.3 antibody recognizes serine 31 of H3.3, which is not present on either H3.1 or H3.2, but cross-reactivity with other histone variants has not been tested experimentally (Abcam).

### Time course and ChIP

For ‘TET-OFF’ experiments, ESCs were cultured over several passages (weeks) on feeder cells in the absence of DOX and were subsequently passaged onto feeder-free plates prior to the inhibition of HA-H3.3 expression. To repress HA/FLAG-H3.3 expression we treated cells with 2 μg/mL doxycycline hyclate before crosslinking with formaldehyde at various time points. ChIP experiments were performed as described previously with an antibody against HA [47]. ChIP-PCR validation was carried out with Rps19 primers (Forward: TTGTCCTCAAGACACCAGTGGAGCT, Reverse: ATCTGCTCAACCGCACTTGG) and with Ichr.8 primers (Forward: AAGGGGCCTCTGCTTAAAAA, Reverse: AGAGCTCCATGGCAGGTAGA).

### Deep sequencing library construction

In order to correlate HA/FLAG-H3.3 stability with the presence of histone modifications and/or gene expression levels, we constructed ChIP-Seq libraries for H2A.Z, H3K4me1, H3K4me3, H3K9ac, H3K27me3, and RNA-Seq libraries.

For RNA-Seq library construction, poly-adenylated RNA was isolated from 5 ug of total RNA isolated from undifferentiated ESCs cultured on gelatin-coated plates, using the Dynabeads mRNA Direct kit (Invitrogen). Double-stranded cDNA was generated with the Double-stranded cDNA synthesis kit (Invitrogen), sonicated, and subsequently processed exactly like ChIP DNA.

ChIP material was blunt-ended and phosphorylated with the End-it-Repair kit (EPICENTRE). Illumina genome sequencing adaptors were ligated with T4 DNA ligase (New England Biolabs) after addition of adenosine nucleotides, using exo-Klenow. Samples were PCR amplified with multiplexed Illumina genomic DNA sequencing primers. PCR products (250 to 450 bp in size) were gel purified and submitted for Illumina deep sequencing.

### Calculation of H3.3 dissociation rates and equilibrium levels

ChIP-Seq reads were mapped to the mouse reference genome (mm9) by perfect and unique matching without allowing any mismatch or gap. The reads were then extended to 150 bp from their 5’ end. We applied a modified average low pass filter for deconvolution of noisy blurred images. The normalized H3.3 level at a 250 bp window is a linear combination of the number of the HA-H3.3 nucleosomes at the site and the number of HA-H3.3 nucleosomes in the two adjacent 250 bp-length windows. In the linear combination, the number of HA-H3.3 nucleosomes at the center is weighted 1 and the number of HA-H3.3 nucleosomes in the two neighborhoods at both ends is weighted 0.5.

For our analysis of H3.3 dissociation rates and equilibrium levels, we only considered genomic regions that show significant enrichment of HA-H3.3 at 0 h of the ‘TET-OFF’ time course and day 6 of the TET-ON time course.

The H3.3 dissociation rate, λ’_*out*_ was calculated following an exponential decay equation:


Where, *N(t)* is amount of H3.3 in a chromatin at time *t* in TET-OFF experiment.

We estimated the H3.3 dissociation rates for any given locus by counting the number of HA-H3.3 nucleosomes mapped at 0 h and 6 h following inhibition of HA-H3.3 expression. To calculate the relative H3.3 dissociation rates at a locus *i* (*λ’*_*out,i*_*)*, we used the following logistic equation to scale dissociation rates to values from 0 to 1:


*N*_*i*_*(0 h)* : the number of HA-H3.3 dyads mapped to locus *i* at 6 h after inhibition of H3.3-HA expression.

*N*_*i*_*(6 h)* : the number of HA-H3.3 dyads mapped to locus *i* at 6 h after inhibition of H3.3-HA expression.

Equilibrium levels of HA-H3.3 at any given locus was estimated from the enrichment of HA-H3.3 at 0 h of TET-OFF experiments.

*N*_*i*_*(0 h)* : the number of HA-H3.3 dyads mapped to locus *i* at 0 h after inhibition of HA-H3.3 expression.

*I*_*i*_*(0 h)* : the number of nucleosome dyads mapped to locus *i* from input DNA sequencing at 0 h after inhibition of HA-H3.3 expression.

### Identification of transcription factor binding sites and chromatin modification peaks

Transcription factor ChIP-Seq for Nanog, Oct4, Sox2, Smad1, E2F1, Tcfcp2I1, CTCF, Zfx, STAT3, KLF4, Esrrb, n-Myc, p300 in mESC were obtained from Chen *et al.* (GSE11431) [48]. H3, H4K20me3 H3K9me3, H3K36me3 ChIP-Seq in mES were obtained from Mikkelsen *et al.* (GSE12241) [15]. KDM2A ChIP-Seq in mESC is obtained from Blackledge *et al.* (GSE21202) [31]. SUZ12, EZH2, RING1B ChIP-Seq in mESC were obtained from Ku *et al.* (GSE13084) [49]. Med12, Smc1/2/3 Med1, Nipbl, CTCF ChIP-Seq in mESC were obtained from Kagey *et al.* [50]. HDAC1, HDAC2, LSD1, REST (transcription repressor of neuronal genes in non-neuronal cells), COREST, Mi2b ChIP-Seq were obtained from Whyte *et al.* (GSE27844) [51]. LMR/UMR/FMR sites in mESC were obtained from Stadler *et al.* [52]. DNaseI hypersensitive sites in mESC were obtained from the Encode project.

The raw ChIP-Seq data in SRA format were transformed into fastq files and mapped to the reference genome (mm9). The 30 to 50 bp sequences from the ChIP-Seq data were mapped to the mouse reference genome (mm9) by perfect and unique matching without allowing any mismatch or gap. The reads were then extended to 150 bp from their 5’ end.

### Analysis of RNA-Seq data

The RNA-Seq analysis was performed using the Tuxedo software package with default settings. RNA-Seq reads were mapped to the mouse genome (NCBI37/mm9) using Bowtie2. Tophat with default settings was used to detect splice sites. The Cufflinks software package was used to assemble transcripts based on the Refseq mRNA sequence database (mm9). A total of 48,228 transcripts were detected from two RNA-Seq replicate experiments and their mean values were used for further analysis. Transcripts were sorted according to their FPKM (fragments per kilobase of exon model per million mapped fragments). The ‘top 10%’ (4,867) of transcripts was defined as having expression levels greater than 30 FPKM, the ‘top 20%’ (4,568) greater than 10 FPKM and less than 30 FPKM, ‘medium’ (5,849) between 3 and 10 FPKM, ‘low’ (4,441) between 1 and 3 FPKM. Silenced transcripts were defined as having expression levels between 0 and 1 FPKM.

### Statistical tests

All statistical tests were performed using the R, stat package. To test the significance of Pearson correlation coefficients, we used the cor.test() in the R stat package. To test the significance of enrichment and depletion, we performed a Chi-squared test using the chisq.test() in the R stat package.

### Data availability

Our ChIP-Seq and RNA-Seq data sets have been deposited in the Gene Expression Omnibus data base with accession number GSE63641.

## Electronic supplementary material

Additional file 1: Figure S1: **(A)** HA-H3.3 ESCs were cultured on feeder cells (upper panel) and cultured for 3 days or seeded at clonal density and grown for 5 days (lower two panels) with and without DOX prior to alkaline phosphatase staining. **(B)** Total RNA was isolated from undifferentiated HA-H3.3 ESCs and from day 8 EBs. Quantitative PCR analysis was performed for analysis of expression of pluripotency factors (Oct4, Nanog, Sox2) as well as differentiation markers (Sox17, Kdr). (PDF 3 MB)

Additional file 2: Figure S2: **(A)** ChIP-Seq profiles of two control regions: Rps19 (positive) and Intergenic Chr.8 (negative) over a 48-h time course after inhibition of HA-H3.3 expression. **(B)** HA-H3.3 ESCs were crosslinked at various time points after DOX addition and ChIP-PCR validation was performed with primers spanning the region highlighted in red. (PDF 1 MB)

## Abbreviations

BP: Base pair
ChIP: Chromain immunoprecipitation
DOX: Doxycycline
EB: Embryoid body
ESC: Embryonic stem cell
HA: Hemagglutinin
MEF: Mouse embryonic fibroblast
Pol: Polymerase
TES: Transcription end site
TET: Tetracycline
TSS: Transcription start site.
