# Supplementary figures and images for: Genome-wide analysis of H3.3 dissociation reveals high nucleosome turnover at distal regulatory regions of embryonic stem cells
Source: Epigenetics Chromatin. 2014 Dec 20;7:38. doi: 10.1186/1756-8935-7-38 (PMC4297464; doi:10.1186/1756-8935-7-38)

Supplementary Figure 1

A

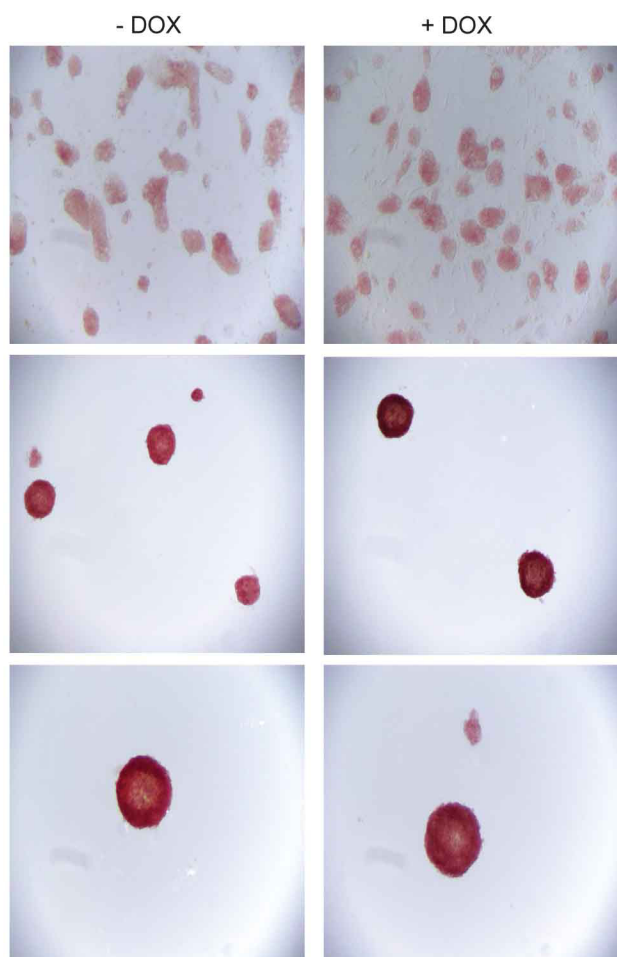

B

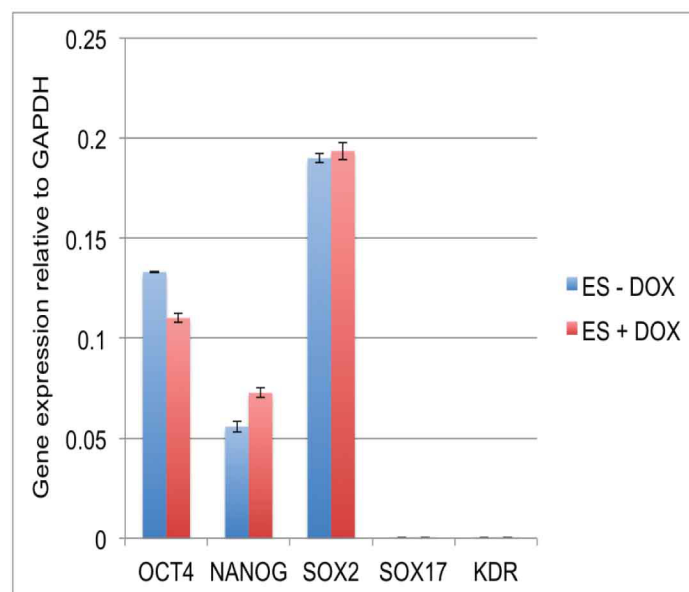

C

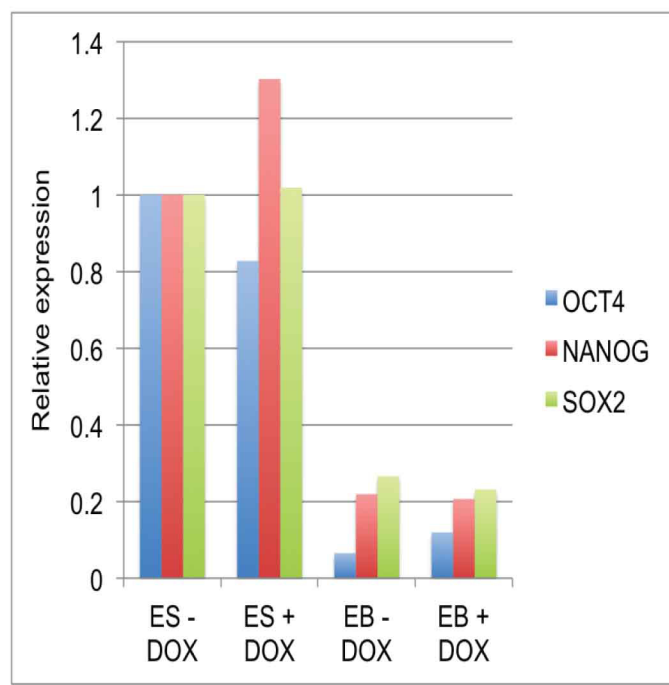

D

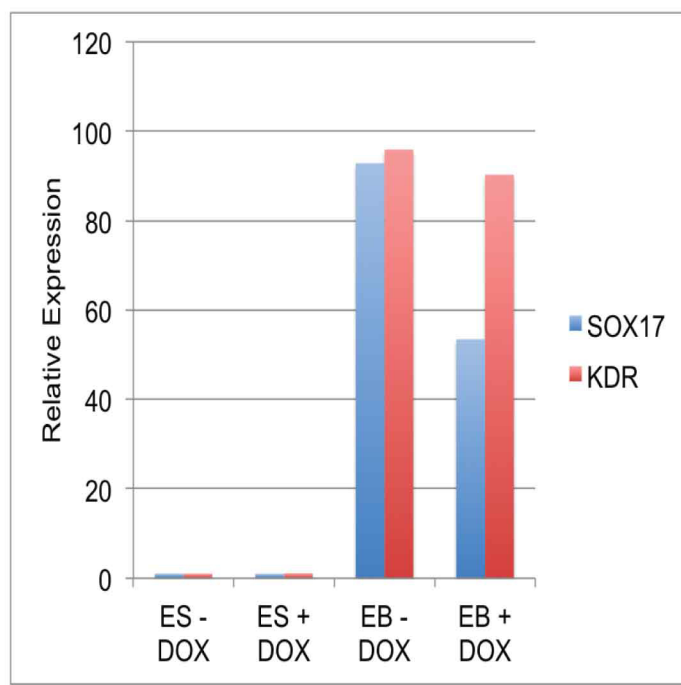

Supplement: Supplementary file 1 — Additional file 1: Figure S1: (A) HA-H3.3 ESCs were cultured on feeder cells (upper panel) and cultured for 3 days or seeded at clonal density and grown for 5 days (lower two panels) with and without DOX prior to alkaline phosphatase staining. (B) Total RNA was isolated from undifferentiated HA-H3.3 ESCs and from day 8 EBs. Quantitative PCR analysis was performed for analysis of expression of pluripotency factors (Oct4, Nanog, Sox2) as well as differentiation markers (Sox17, Kdr). (PDF 3 MB) [file 13072_2014_344_MOESM1_ESM.pdf]

Supplementary Figure 2

A

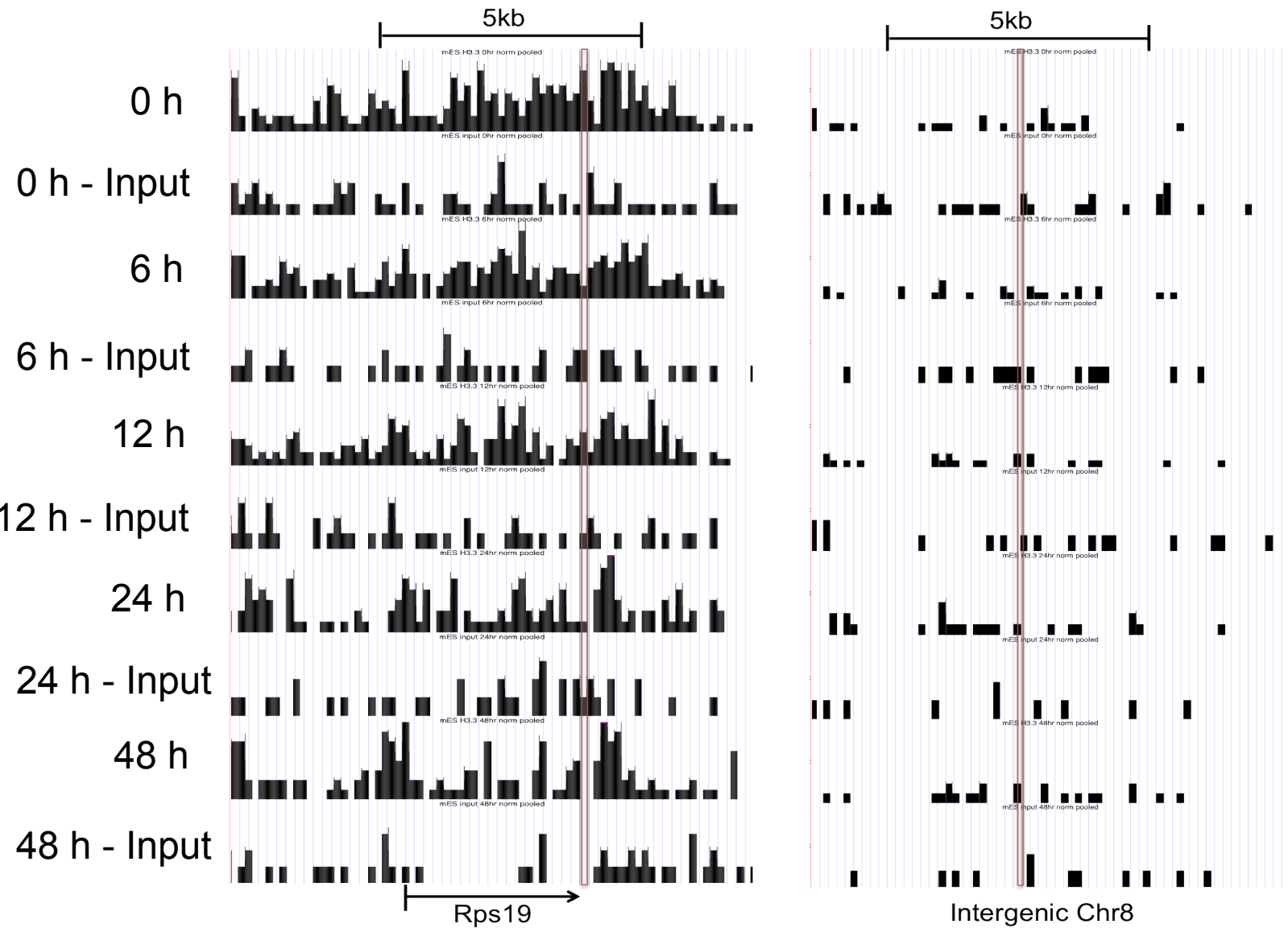

B

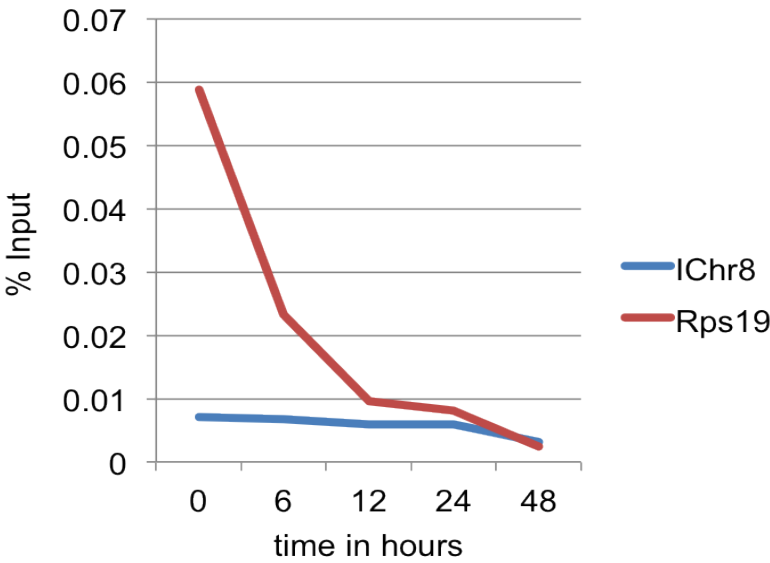

Supplement: Supplementary file 2 — Additional file 2: Figure S2: (A) ChIP-Seq profiles of two control regions: Rps19 (positive) and Intergenic Chr.8 (negative) over a 48-h time course after inhibition of HA-H3.3 expression. (B) HA-H3.3 ESCs were crosslinked at various time points after DOX addition and ChIP-PCR validation was performed with primers spanning the region highlighted in red. (PDF 1 MB) [file 13072_2014_344_MOESM2_ESM.pdf]
